# Data of cost-optimality and technical solutions for high energy performance buildings in warm climate

**DOI:** 10.1016/j.dib.2015.05.015

**Published:** 2015-06-12

**Authors:** Ilaria Zacà, Delia D’Agostino, Paolo Maria Congedo, Cristina Baglivo

**Affiliations:** a Department of Engineering for Innovation, University of Salento, Lecce 73100, Italy; b Energy Efficiency and Renewables Unit, Institute for Energy and Transport (IET), Joint Research Centre (JRC), European Commission, Ispra, Varese, Italy

## Abstract

The data reported in this article refers to input and output information related to the research articles entitled Assessment of cost-optimality and technical solutions in high performance multi-residential buildings in the Mediterranean area by Zacà et al. (Assessment of cost-optimality and technical solutions in high performance multi-residential buildings in the Mediterranean area, in press.) and related to the research article Cost-optimal analysis and technical comparison between standard and high efficient mono residential buildings in a warm climate by Baglivo et al. (Energy, 2015, 10.1016/j.energy.2015.02.062, in press).

The European policy framework is focused on reducing energy consumption in the building sector. The recast of EU (European Union) Directive on EPBD (Energy Performance of Buildings) requires nZEBs (nearly zero energy buildings) as the building target from 2018 onwards and the establishment of cost optimal levels of minimum energy performance requirements in buildings.Zacà et al. [1] shows a methodology to assess energy and cost effectiveness in new buildings located in the Mediterranean area. Several energy efficiency technical variants are applied to a multi residential reference building selected as a representative model of the national building stock.Baglivo et al. [2] presents the results of the application of a methodology to identify cost-optimal levels in new residential buildings located in a warm climate. Mono-residential buildings have been considered as virtual reference buildings in this study. Different energy efficiency measures have been selected for the envelope and the systems.

High performance of different solutions can be obtained in summertime by using lighter and thinner walls, although the superficial mass of the external wall is important to obtain the best performance in the hot-summer Mediterranean climate [3–5]. Heat transfer through windows represents a significant proportion of the energy used to cover both heating and cooling requirements [6]. Data related to building input values are supplied for mono and multi-family units and the main values of HVAC and RES systems are provided. The decision to introduce HVAC [7] and RES systems [8,9] as variants derived from the necessity to reduce energy consumption maintaining a high level of comfort in all the building rooms.

**Specifications table**

**Table:** 

Subject area	*Physics*
More specific subject area	*Describe narrower subject area*
Type of data	*Table*
How data was acquired	*TABULA project***(**Building typology brochure Italy) [10,11]*, technical datasheets*[4]*, Puglia price list, market surveys, ISTAT surveys, software ProCasaClima 2015*[12]
Data format	*Raw*
Experimental factors	*No pretreatment of samples was performed*
Experimental features	*Several energy efficiency technical variants are applied to residential reference buildings selected as representative model of the national building stock. Primary energy consumption and global costs are evaluated in a number of configurations to derive the cost-optimal solution.*
Data source location	*Lecce—Italy, Mediterranean climate*[3]
Data accessibility	*Data is provided in Supplementary materials directly with this article*

**Value of the data**

• A methodology is applied to obtain cost-optimal levels in a residential reference buildings located in a warm climate.
• Energy performance is assessed for different packages of energy efficiency technical measures.
• Global costs are evaluated to identify the cost-optimal solution.

**Data, experimental design, materials and methods**

The data in this data article has been generated by following these main steps:

1) Definition of the reference buildings and characterization of its envelope and systems;
2) Establishment of technical variants and combinations for energy performance assessment;
3) Global costs calculations.

This method allowed the identification of measures able to optimize the energy performance of the selected reference buildings. Cost-optimal and cost effective levels are finally derived and discussed for the cases study.

The reference buildings are based on solutions defined within TABULA project (virtual buildings) (Table S1), including commonly used material and systems. The location is the city of Lecce—Italy, characterized by a Mediterranean climate.

Heating and cooling loads are obtained using the software ProCasaClima2015. The developed tool uses hourly weather data provided by the Italian Heat Technology Committee to implement dynamic simulations. This software is able to perform energy calculations to evaluate buildings energy requirements in compliance with Directives 2010/31/EU and 2012/27/EU. It is necessary to enter the characteristics of the building site, envelope and systems to obtain numerical and graphical outputs, such as winter and summer energy demand for CasaClima certification or thermal energy demand in summer and winter (UNI TS 11300-1, UNI TS 11300-2) [12].

ProCasaClima2015 can derive costs and benefits of possible interventions (UNI EN 15459) [13]. Energy requirements for cooling and dehumidification can be derived with the estimation of indoor comfort without active cooling and dehumidification.

A proper selection of energy efficiency technological measures are able to reduce considerably the energy needs of a building. Building envelope is a key element to decrease the energy demand.

The definition of external walls as an optimized multilayer package has been obtained through the integration of a multi-criteria optimization analysis [4,5] (Table S2).

The cooling energy performance is estimated for different fenestration systems considering various combinations of thermal transmittance, *U*-value in different conditions, orientation, and shading. The solar transmittance of the whole window depends on the area of the transparent element (Table S3).

HVAC systems are evaluated to satisfy the demand of thermal comfort, ventilation and DHW production. Table S4 shows technical systems for building configurations, each one including RES. All systems are designed to obtain an internal temperature of 26 °C with 50% relative humidity (RH) in summer, and 20 °C with 50% RH in winter.

A combination of technical variants has been then applied to the reference case in order to obtain several configurations to be compared in terms of primary energy consumption and global costs (Table S5). The cost-optimal solution is identified assessing technical features and energy performance. Standard and high efficiency buildings are analyzed to show how the selected configuration allows a decrease in primary energy consumption and CO_2_ emissions at the lowest cost. Results are useful for comparison with other climates and building types. They also show the feasibility of the methodology to comply with EU requirements and to support the choice of economically efficient nZEBs solutions at the design stage.
